# Dual blockade of DPP-4 and CXCL12/CXCR4 axes synergistically protects podocytes in lupus nephritis

**DOI:** 10.3389/fphar.2025.1732243

**Published:** 2026-01-12

**Authors:** Hui-miao Hu, Yong-chun Li, Yang-ming Zhang, Teng-yu Zhu, Wei-jing Yong, Yu-hui Gan, Chen Liu, Rui-ying Duan, Hong-de Xu, Zhan-zheng Zhao, Yuan-yuan Qi

**Affiliations:** 1 Department of Nephrology, The First Affiliated Hospital of Zhengzhou University, Zhengzhou, China; 2 Zhengzhou University, Zhengzhou, China; 3 Ministry of Education of China, Institute of Drug Discovery and Development, Zhengzhou University, Zhengzhou, China; 4 School of Pharmaceutical Sciences, Zhengzhou University, Zhengzhou, China; 5 Laboratory of Nephrology, The First Affiliated Hospital of Zhengzhou University, Zhengzhou, China

**Keywords:** CXCL12/CXCR4 axis, dipeptidyl peptidase-4 inhibitor, lupus nephritis, podocytes, renoprotection

## Abstract

**Background:**

Lupus nephritis (LN), a severe complication of systemic lupus erythematosus (SLE), is characterized by podocyte injury that contributes to disease progression. Dipeptidyl peptidase-4 (DPP-4) inhibitors, though developed for diabetes, have shown renoprotective potential. However, DPP-4 inhibition may elevate CXCL12/CXCR4 signaling, a pathway implicated in LN pathogenesis. This study aimed to determine whether dual DPP-4 and CXCL12/CXCR4 blockade confers enhanced renal protection in LN.

**Methods:**

MRL/lpr lupus-prone mice were treated with the DPP-4 inhibitor linagliptin, either alone or in combination with the CXCL12/CXCR4 axis antagonist AMD3100. We evaluated renal function, histopathology, podocyte structure, and markers of oxidative stress, fibrosis, and inflammation. *In vitro* assays using DPP4-knockout podocytes were also performed to elucidate underlying mechanisms.

**Results:**

DPP4-deficient podocytes exhibited elevated CXCL12/CXCR4 expression and modest nephrin upregulation. Co-treatment with AMD3100 further increased nephrin expression compared to linagliptin alone. *In vivo*, linagliptin monotherapy reduced proteinuria and serum creatinine but also increased CXCL12/CXCR4 expression. Combined therapy significantly decreased proteinuria, serum creatinine, anti-dsDNA, and ANA titers. Histological analysis showed reduced mesangial proliferation and interstitial inflammation. Transmission electron microscopy and immunostaining demonstrated improved podocyte foot process integrity and upregulation of nephrin and podocin. Dual blockade also reduced renal oxidative stress (DHE, NOX4), fibrosis markers (α-SMA, fibronectin), and inflammatory mediators (NF-κB p65, NLRP3).

**Conclusion:**

Combined DPP-4 and CXCL12/CXCR4 axis inhibition synergistically enhanced podocyte protection and attenuated renal inflammation, fibrosis, and oxidative stress in lupus nephritis. These findings support dual blockade as a promising therapeutic strategy for LN.

## Introduction

Systemic lupus erythematosus (SLE) is an autoimmune disease that affects multiple organs and systems, among which lupus nephritis (LN) is considered one of the most severe manifestations with a poor prognosis (Kaul et al., 2016; Anders et al., 2020). The clinical course and pathological features of LN are markedly heterogeneous; despite standard immunosuppressive therapy, some patients may gradually progress to end-stage renal disease due to persistent glomerular and interstitial injury (Conrad et al., 2023). Studies have confirmed that podocyte injury is ubiquitous in the pathogenesis of LN, and structural and functional abnormalities in podocytes can lead to proteinuria, thereby accelerating the onset and progression of glomerular dysfunction (Yu et al., 2017; Clark and Greka, 2020). Thus, elucidating the molecular mechanisms underlying podocyte injury is of great theoretical and clinical significance for developing novel intervention strategies to delay renal deterioration.

Dipeptidyl peptidase-4 (DPP-4) inhibitors were initially developed for the treatment of type 2 diabetes mellitus (T2DM), with their primary mechanism of action being the prolongation of the half-life of glucagon-like peptide-1 (GLP-1), thereby enhancing insulin secretion and improving glycemic control (Deacon, 2020). In recent years, an increasing number of studies have focused on the potential renoprotective effects of DPP-4 inhibitors in T2DM-related kidney disease. The 2019 CARMELINA trial demonstrated that linagliptin could delay the progression of proteinuria, although its effect on composite renal endpoints was not statistically significant (Rosenstock et al., 2019). Meta-analyses have indicated that linagliptin in combination with Angiotensin-Converting Enzyme Inhibitors (ACEI) or angiotensin receptor blockers (ARB) can significantly reduce urinary albumin levels and lower the overall risk of adverse renal events by approximately 16% (Cooper et al., 2015). Furthermore, subgroup analyses from the SAVOR-TIMI 53 trial suggested that saxagliptin may reduce proteinuria, yet its impact on estimated glomerular filtration rate (eGFR) was not significantly different from that of placebo (Mosenzon et al., 2017). The MARLINA-T2D study, which was the first clinical trial to evaluate the effects of linagliptin on renal outcomes in T2DM patients with proteinuria on top of standard therapy, failed to demonstrate a significant reduction in urinary albumin levels (Groop et al., 2017). Overall, although preliminary data support a certain degree of renoprotection by DPP-4 inhibitors in T2DM-associated kidney disease, controversies remain, and large-scale, multicenter randomized controlled trials with renal outcomes as the primary endpoint are needed to further establish their long-term efficacy.

In recent years, increasing attention has also been directed toward the potential protective effects of DPP-4 inhibitors in non-diabetic kidney diseases. Animal studies have revealed that DPP-4 inhibitors can significantly reduce oxidative stress in renal tissues, suppress local inflammatory responses, and attenuate interstitial and glomerular fibrosis, thereby improving renal function and delaying disease progression (Liu et al., 2012; Tsuprykov et al., 2016). However, recent findings suggest that during treatment with DPP-4 inhibitors, the CXCL12/CXCR4 signaling axis may become compensatory or aberrantly upregulated (Li et al., 2020; Kawakita et al., 2023).

At the molecular level, DPP-4 (CD26) cleaves the N-terminal two amino acids of CXCL12 (also known as SDF-1α), converting full-length CXCL12 into a truncated form with markedly reduced chemotactic activity (Wang et al., 2014). Conversely, the use of DPP-4 inhibitors blocks this cleavage, leading to elevated *in vivo* levels of full-length CXCL12 and enhanced binding to its receptor, CXCR4. Previous studies have demonstrated that the CXCL12/CXCR4 signaling axis plays a critical role in the pathogenesis of SLE, suggesting that targeting this pathway may offer a novel therapeutic strategy for improving SLE outcomes (Chong and Mohan, 2009). For instance, one study reported that serum CXCL12 levels were significantly elevated in SLE patients (Robak et al., 2007), while the expression of CXCR4 on peripheral B cells and CD4^+^ T cells was also markedly upregulated and correlated with disease activity (Wang et al., 2010). Moreover, in renal tissues of LN patients, CXCL12 expression is abnormally increased, which may promote the recruitment of CXCR4^+^ immune cells to the kidney and thereby exacerbate local inflammation and tissue damage (Wang et al., 2010). In addition to B cells and monocytes, CXCR4 is also highly expressed on CD4^+^ and CD8^+^ T cells in both lupus patients and lupus-prone mice, where it contributes to their aberrant activation and renal infiltration (Wang et al., 2010; Dow et al., 2013). In animal models, lupus-prone mice exhibit high CXCR4 expression in B cells, monocytes, neutrophils, and plasma cells, along with significantly elevated CXCL12 levels in the glomeruli and tubules (Wang et al., 2009). Furthermore, in lupus-prone NZB/W mice, increased renal CXCL12 expression is closely associated with nephritis and autoantibody production, and administration of anti-CXCL12 monoclonal antibodies has been shown to effectively prevent the development of nephritis (Balabanian et al., 2003).

Given that DPP-4 inhibitors display renoprotective effects by reducing oxidative stress, inflammation, and fibrosis, their use may paradoxically induce upregulation of the CXCL12/CXCR4 signaling axis, which could further exacerbate the recruitment of immune cells and local inflammatory responses. Therefore, we propose a novel therapeutic strategy that combines DPP-4 inhibitors with CXCL12/CXCR4 axis inhibitors. This combined treatment regimen aims to fully harness the antioxidant, anti-inflammatory, and antifibrotic benefits of DPP-4 inhibitors, while simultaneously inhibiting the CXCL12/CXCR4 signaling pathway to effectively block aberrant immune cell recruitment to the kidney, thereby protecting podocytes and ultimately improving renal injury in patients with lupus nephritis.

## Methods

### Animal models and treatments

MRL/lpr mice are a classic spontaneous lupus nephritis model, characterized by progressive systemic autoimmunity with profound lymphoproliferation, accumulation of autoreactive CD4^+^ and CD8^+^ T cells, expansion of abnormal B-cell subsets, and markedly elevated autoantibody production, ultimately leading to overt nephritis. Female mice were utilized in this study, as they exhibit more consistent, robust, and reproducible disease phenotypes. Female MRL/lpr mice (Jiangsu Huachuang Xinnuo, China) were housed under standard SPF conditions. At 16 weeks of age, mice were treated for 4 weeks with linagliptin (3 mg/kg) or losartan (10 mg/kg) via oral gavage, or AMD3100 (3 mg/kg) via intraperitoneal injection. Control mice received saline. After treatment, 24-h urine samples were collected, and mice were sacrificed for serum and kidney tissue collection. Mice were euthanized under deep isoflurane anesthesia prior to tissue collection. All procedures were approved by the Ethics Committee of Zhengzhou University (ZZU-LAC20230220).

### Histopathological examination

Kidneys were fixed, sectioned, and stained with hematoxylin and eosin (H&E), periodic acid-Schiff (PAS), and Masson. Paraffin sections were cut at 3–4 μm thickness for histological staining. Transmission electron microscopy (TEM) was performed for ultrastructural evaluation. All assessments were conducted by board-certified renal pathologists blinded to group allocation.

### Biochemical measurements

Serum and urine samples were analyzed for creatinine, urinary protein, anti-dsDNA IgG, and ANA levels using commercial kit, following manufacturers’ protocols. Creatinine and urinary protein were measured using specific kits (Cat#C035-2-1; Cat#C011-2-1, Nanjing Jiancheng Bioengineering Institute). Anti-dsDNA IgG was quantified using Mouse Anti-dsDNA IgG ELISA Kit (Cat#ADI-5110, Alpha Diagnostic International). For the determination of ANA, we used the Mouse Anti-Nuclear Antibodies (ANA) Total Ig ELISA Kit (Cat#ADI-5210, Alpha Diagnostic International).

### Generation of CRISPR/Cas9 cell lines

To generate DPP4-knockout podocytes, four sgRNAs targeting human DPP4 were designed: sgRNA1:5′-CACCATCATCACCGTGCCCG-3′; sgRNA2:5′-AACCACGGGCACGGTGATGA-3′; sgRNA3:5′-CCCGTGGTTCTGCTGAACAA-3′; sgRNA4:5′-CCTTTGTTCAGCAGAACCAC-3′. After experimental verification, we ultimately used sgRNA2:5′- ACCACGGGCACGGTGA-3′ to construct a knockout cell line. The sgRNAs were cloned into a Cas9-expressing vector and transfected into podocytes (1 × 10^6^ cells) using Lipofectamine™ 3000 (Thermo Fisher Scientific, Waltham, Massachusetts, United States). At 72 h post-transfection, cells were selected with puromycin (1 μg/mL) for an additional 72 h. The surviving cell population was expanded and subjected to single-cell cloning. Genomic DNA was subsequently isolated, and DPP4 knockout was validated by PCR.

### Cell culture and differentiation

Conditionally immortalized human podocytes were propagated at 33 °C (permissive temperature) in RPMI 1640 medium supplemented with 10% heat-inactivated fetal bovine serum (FBS) and 1× Insulin-Transferrin-Selenium (ITS; Gibco, 41,400-045) in a humidified 5% CO_2_ atmosphere. To induce differentiation, podocytes were seeded at the required density and transferred to 37 °C (non-permissive temperature) for 10–14 days. During the differentiation period, cells were maintained in RPMI 1640 medium containing 10% FBS with medium changes every 2–3 days.

### Plasmid overexpression and transfection

The human DPP4 overexpression plasmid and corresponding empty vector (Vigene Biosciences Inc., Shandong, China) were amplified in *E. coli* Stbl3 (TransGen Biotech, Beijing, China) and purified using an endotoxin-free maxi-prep kit (Qiagen). Fully differentiated human podocytes were seeded in 6-well plates (2.5 × 10^5^ cells/well) 24 h before transfection with 2.5 μg plasmid using Lipofectamine™ 3000 (Thermo Fisher Scientific) according to the manufacturer’s protocol. Medium was replaced 6–8 h later, and cells were harvested 48–72 h post-transfection for subsequent analyses.

### Isolation of immunoglobulin G from serum samples

Patient-derived IgG was used in in vitro experiments to mimic immune-complex–mediated podocyte injury, a key pathogenic mechanism in lupus nephritis. Serum IgG was isolated from SLE patients using a Protein A-based affinity purification system. Whole blood was collected in BD Vacutainer® tubes and centrifuged at 3,000 × g for 15 min. IgG was purified according to a previously published protocol (Yuan-yuan, 2018), aliquoted, and stored at −80 °C. The study protocol was approved by the Ethics Committee of the First Affiliated Hospital of Zhengzhou University. (approval number:2019-KY-134).

### Western blot

A total of 20–30 μg protein per lane was loaded for SDS-PAGE, and electrophoresis was performed under standard reducing conditions. Proteins from podocytes and renal tissues were extracted, separated by SDS-PAGE, and transferred to PVDF membranes. Membranes were blocked with 5% skim milk, incubated with primary antibodies overnight at 4 °C, and then with HRP-conjugated secondary antibodies (1:10,000) for 2 h at room temperature. Detection was performed using ECL reagents and imaged on an automated system. Densitometry was performed using ImageJ (v1.53).

Primary antibodies: anti-SDF1 (ab25117, Abcam), anti-CXCR4 (ab181020, Abcam), anti-NPHS2 (ab50339, Abcam), anti-nephrin (ab58968, Abcam), anti-α-tubulin (2144S, Cell Signaling Technology), anti-p65 (8242S, Cell Signaling Technology), anti-NLRP3 (ab263899, Abcam), anti-caspase-1 (PA5-87536, ThermoFisher Scientific), anti-cleaved caspase-1 (PA5-38099, ThermoFisher Scientific), anti-IL-1β (ab283822, Abcam), anti-α-SMA (ab5694, Abcam), anti-fibronectin (ab2413, Abcam). Secondary antibody: HRP-conjugated goat anti-rabbit IgG (GB23301 and GB23303, Servicebio).

### Dihydroethidium staining

Fresh-frozen kidney sections were incubated with 5 μM DHE (HY-D0039, MedChemExpress) in PBS for 45 min at 37 °C in the dark. After washing, nuclei were counterstained with DAPI (G1012, Servicebio). Images were acquired with a fluorescence microscope.

### Immunofluorescence staining

Tissue sections were fixed, permeabilized, and blocked with 3% BSA (GC305010, Servicebio). Sections were incubated overnight at 4 °C with primary antibodies against SDF1 (ab25117, Abcam), synaptopodin (sc-515842, SANTA CRUZ BIOTECHNOLOGY), IgG (ab172730, Abcam), NOX4 (ABC459, Sigma-Aldrich), and WT1 (ab89901, Abcam). After PBS washes, fluorescent-conjugated secondary antibodies were applied. Nuclei were counterstained with DAPI (G1012, Servicebio) and slides mounted with antifade medium. Images were captured using a fluorescence microscope.

### Immunohistochemical staining

Paraffin-embedded renal sections were fixed in 4% paraformaldehyde, subjected to citrate-based antigen retrieval (G1201, Servicebio), and treated with 3% H2O2. After blocking with 3% BSA (GC305010, Servicebio), sections were incubated overnight at 4 °C with antibodies against α-SMA (ab5694, Abcam) and fibronectin (ab2413, Abcam). HRP-conjugated secondary antibodies (GB23301 and GB23303, Servicebio) were applied, followed by color development with DAB (G1211, Servicebio). Slides were visualized using bright-field microscopy.

### Statistical analysis

Data analyses and graphical visualizations were performed using GraphPad Prism 9. Intergroup comparisons were performed using one way ANOVA with Tukey’s multiple comparison test. The exact sample size (n) for each group—representing biologically independent animals for *in vivo* experiments or independent replicate experiments for *in vitro* studies—is provided in the respective figure legends. Statistical significance was defined as P < 0.05. Error bars represent standard deviation (SD).

## Results

### DPP4 inhibitor linagliptin mitigates symptoms of lupus nephritis

In the MRL/lpr lupus mouse model, we evaluated the therapeutic efficacy of the DPP4 inhibitor linagliptin. Mice were randomly allocated into three groups: control, losartan, and linagliptin treatment groups. Linagliptin administration did not result in significant alterations in ANA or dsDNA levels (Supplementary Appendix Figure 1A,B). However, it led to a substantial reduction in urinary protein excretion (Supplementary Appendix Figure 1D), although the losartan-treated group exhibited a more pronounced decrease in urinary protein levels. Additionally, serum creatinine concentrations were significantly lower in the linagliptin group compared to the control group, with the losartan group demonstrating the most substantial reduction (Supplementary Appendix Figure 1C).

Histopathological examination of renal tissues revealed that both linagliptin and losartan treatments significantly attenuated mesangial cell proliferation (Supplementary Appendix Figure 1E). Transmission electron microscopy further corroborated these findings, showing that treatment with linagliptin and losartan markedly decreased podocyte foot process fusion, thereby indicating preservation of podocyte architecture (Supplementary Appendix Figure 1E).

### CXCL12 is upregulated in lupus nephritis and correlates with podocyte injury

MRL/lpr mice are an established spontaneous model for lupus nephritis. Our previous study demonstrated the expected progression from minimal renal injury at 8 weeks to overt nephritis at 20 weeks in MRL/lpr mice, reflecting the natural course of disease in this model (Lv et al., 2022). To elucidate the role of CXCL12/CXCR4 axis in lupus nephritis, we assessed CXCL12 expression in renal tissues at different disease stages. Immunofluorescence microscopy analysis revealed a significant upregulation of CXCL12 in podocytes of 20-week-old MRL/lpr mice compared to 8-week-old mice (Supplementary Appendix Figure 2A). This elevation in CXCL12 protein levels was further confirmed by Western blot analysis (Supplementary Appendix Figure 2B).

The expression of CXCL12, CXCR4 and nephrin was upregulated in MRL/lpr mice treated with linagliptin or losartan, the linagliptin group showing a more significant increase (Supplementary Appendix Figure 2C).

### Dual inhibition of DPP4 and CXCL12/CXCR4 axis enhances podocyte protection in vitro

*In vitro* experiments were conducted using a DPP4 knockout podocyte cell line. Compared to control cells, DPP4-deficient podocytes displayed significantly elevated expression of CXCL12 and CXCR4, alongside increased nephrin levels (Supplementary Appendix Figure 2D). Treatment with the DPP4 inhibitor further augmented the expression of CXCL12, CXCR4, and nephrin (Supplementary Appendix Figure 2E).

Based on these observations, we hypothesized that CXCL12/CXCR4 axis upregulation may negatively impact podocyte integrity. To test this hypothesis, we implemented a dual inhibition strategy targeting DPP4 and CXCL12/CXCR4 axis using linagliptin and AMD3100, respectively. The dual blockade resulted in a significantly higher expression of nephrin compared to treatment with the DPP4 inhibitor alone, indicating enhanced podocyte protection (Supplementary Appendix Figure 2F).

### Dual inhibition of DPP4 and CXCL12/CXCR4 axis exerts superior therapeutic effects in lupus nephritis mouse models

*In vivo* studies involved treating 20-week-old MRL/lpr mice with either linagliptin alone or in combination with AMD3100. The dual inhibition group exhibited significantly reduced levels of ANA and dsDNA, as well as further decreases in urinary protein and serum creatinine compared to the linagliptin monotherapy group (Supplementary Appendix Figure 3A–D).

Histopathological assessment indicated that dual inhibition significantly decreased mesangial cell proliferation and interstitial inflammatory infiltration relative to linagliptin treatment alone (Supplementary Appendix Figure 3E). Immunofluorescence analysis revealed a marked reduction in IgG deposition within the glomeruli in the dual inhibition group (Supplementary Appendix Figure 2F).

Podocyte integrity was further evaluated by transmission electron microscopy, which demonstrated that dual inhibition more effectively reduced podocyte foot process fusion, thereby better preserving podocyte structure (Supplementary Appendix Figure 3F). Additionally, immunofluorescence staining for podocyte-specific markers WT1 and synaptopodin showed increased expression levels in the linagliptin group, with further elevation observed in the dual inhibition group (Supplementary Appendix Figure 3F). Western blot analysis corroborated these findings, revealing increased expression of nephrin and podocin—critical structural proteins of podocytes—in both linagliptin and dual inhibition groups, with the dual inhibition group showing significantly higher levels (Supplementary Appendix Figure 3G).

### Dual inhibition of DPP4 and CXCL12/CXCR4 axis attenuates oxidative stress, reduces renal fibrosis, and suppresses inflammatory responses

Lupus nephritis is characterized by heightened renal oxidative stress (Moroni et al., 2010). To evaluate the impact of dual DPP4 and CXCL12/CXCR4 axis inhibition on oxidative status, we assessed DHE and NOX4 expression. DHE is a fluorescent probe for detecting ROS, and NOX4 is a key enzyme involved in ROS production. Linagliptin treatment significantly reduced DHE and NOX4 expression (Supplementary Appendix Figure 4A), with dual inhibition further decreasing their levels (Supplementary Appendix Figure 4A).

Fibrotic changes were assessed by measuring FN and α-SMA expression, both established markers of renal fibrosis. Linagliptin treatment resulted in decreased FN and α-SMA levels (Supplementary Appendix Figure 4B), and these reductions were more pronounced in the dual inhibition group (Supplementary Appendix Figure 4B).

Inflammatory responses were evaluated by quantifying the expression of NF-κB p65 and NLRP3 inflammasome, both pivotal in lupus nephritis pathogenesis. Linagliptin treatment led to reduced expression of NF-κB p65 and NLRP3 (Supplementary Appendix Figure 4C), and dual inhibition further suppressed their expression levels (Supplementary Appendix Figure 4C).

## Discussion

In the present study, we utilized the MRL/lpr lupus mouse model to evaluate the effects of the DPP-4 inhibitor linagliptin as monotherapy and in combination with blockade of the CXCL12/CXCR4 signaling axis on LN. The experimental results demonstrated that treatment with linagliptin alone significantly reduced proteinuria and serum creatinine levels, indicating a degree of renoprotection. Further analyses revealed that during linagliptin treatment, the expression of the CXCL12/CXCR4 signaling axis in renal tissue was markedly upregulated. Both *in vitro* and *in vivo* experiments showed that combined treatment with linagliptin and CXCL12/CXCR4 pathway inhibitors resulted in higher expression levels of key podocyte structural proteins, along with further improvements in various renal function parameters compared with the monotherapy group. These findings provide preliminary evidence that a combined intervention strategy may more effectively ameliorate LN-associated renal changes, thus offering a basis for further optimization of therapeutic regimens.

Our study confirmed the renoprotective effects of linagliptin monotherapy in the MRL/lpr lupus mouse model, a finding consistent with previous reports. For instance, Liu et al. demonstrated in a diabetic rat model that the DPP-4 inhibitor LAF237 significantly reduced urinary albumin levels and alleviated glomerular sclerosis and basement membrane thickening (Liu et al., 2012), while in a 5/6 nephrectomized non-diabetic rat model, linagliptin significantly attenuated interstitial fibrosis and proteinuria (Tsuprykov et al., 2016). Earlier studies have provided important evidence for understanding the protective effects of DPP-4 inhibitors in LN (Li et al., 2024). These investigations primarily focused on the role of DPP-4 inhibitors in reducing renal injury, mitigating mesangial cell abnormalities, and improving renal histological architecture. However, our study further detected a marked upregulation of CXCL12 and its receptor CXCR4 in the renal tissue during linagliptin monotherapy. This observation suggests that while DPP-4 inhibitors confer renoprotection, their use may concurrently enhance CXCL12/CXCR4 signaling, potentially counteracting some of the beneficial effects on the kidney.

To further validate this phenomenon, we treated podocytes *in vitro* with the DPP-4 inhibitor. The results showed that both CXCL12 and CXCR4 expression were significantly increased in podocytes following DPP-4 inhibition. Although treatment with the DPP-4 inhibitor alone moderately increased the expression of nephrin, a key podocyte structural protein, combined blockade of the CXCL12/CXCR4 axis led to a more pronounced upregulation of nephrin. This indicates that the combined intervention further enhances podocyte protection. In animal experiments, the combination therapy group exhibited significantly greater reductions in proteinuria and serum creatinine levels compared with the monotherapy group. Notably, in the combination group, markers of inflammation and oxidative stress—including NF-κB p65, the NLRP3 inflammasome, NOX4, and DHE staining—were significantly decreased, suggesting that the dual intervention more effectively mitigates inflammation and oxidative damage. Although downstream signaling of CXCL12/CXCR4 was not directly examined in this study, our *in vivo* and *in vitro* results collectively suggest that activation of the CXCR4 axis contributes to podocyte injury. The observed reduction in NF-κB p65, NLRP3 inflammasome components, oxidative stress markers, and the restoration of nephrin/podocin expression after AMD3100 treatment closely mirror previously reported CXCR4-mediated activation of β-catenin, JAK/STAT, and NF-κB pathways in podocyte injury models (Mo et al., 2017; Mo et al., 2022; Liu et al., 2020). These parallels support a mechanistic link between CXCL12/CXCR4 upregulation and podocyte damage, even in the absence of additional pathway-specific signaling assays in the present study.

CXCL12 is a key chemokine whose primary receptor, CXCR4, belongs to the G protein-coupled receptor family. This signaling axis plays a central role in regulating cell chemotaxis, hematopoiesis, organ development, and tissue repair, and its aberrant activation is closely associated with the pathogenesis of various pathological conditions, including autoimmune diseases and malignancies (Seppe et al., 2023). In LN patients and corresponding animal models, several studies have reported elevated levels of CXCL12 in renal tissues, along with increased expression of CXCR4 on peripheral and local immune cells (Wang et al., 2009; Wang et al., 2010). This abnormal expression pattern may be closely related to aberrant immune cell recruitment and sustained inflammatory responses, potentially leading to further renal impairment (Launay et al., 2013; Cheng et al., 2018). In an adriamycin (ADR)-induced nephropathy model, increased CXCR4 expression was found to be associated with podocyte injury, and blockade of this pathway reduced podocyte oxidative stress, proteinuria, and fibrosis (Mo et al., 2022). Inhibition of CXCR4 has been shown to mitigate renal inflammation and fibrosis by suppressing downstream signaling pathways—including β-arrestin-1/Src, ERK1/2, GSK-3β, and the CXCR4-AT1 axis (Tang et al., 2022; Qinyu et al., 2024)—while CXCR4 signaling may also activate the JAK/STAT/GSK3β/β-catenin pathway, thereby exacerbating tubular injury and fibrosis (Liu et al., 2020). In contrast, CXCL12 exhibits a dual role in renal diseases; some studies have demonstrated that CXCL12 blockade increases podocyte numbers, reduces proteinuria, and promotes glomerular repair (Romoli et al., 2018). Moreover, DPP-4 inhibition can protect the diabetic kidney by enhancing SDF-1-dependent antioxidant and antifibrotic effects, as well as by improving adverse renal hemodynamics (Takashima et al., 2016). Thus, whether CXCL12 exerts protective or injurious effects in renal disease appears to be highly dependent on the specific disease model and pathological stage, emphasizing the need for comprehensive evaluation in clinical settings.

We also acknowledge certain limitations in our study. In evaluating the combined drug strategy, it is essential not only to assess the individual therapeutic effects of each agent but also to conduct a thorough evaluation of their pharmacokinetics and pharmacodynamics to determine whether significant drug-drug interactions exist. This study was performed exclusively in female MRL/lpr mice, which are commonly used due to their earlier and more consistent lupus nephritis onset. Male mice were not assessed, and potential sex-specific differences could not be evaluated, representing a limitation of the current work.

In summary, our study demonstrates that linagliptin monotherapy exerts a certain degree of renoprotection in the treatment of lupus nephritis, but it also reveals that this treatment is accompanied by an upregulation of the CXCL12/CXCR4 signaling axis, which may potentially compromise renal protection. In contrast, combined blockade of the CXCL12/CXCR4 axis significantly improved renal function, alleviated inflammation, fibrosis, and oxidative stress, and provided enhanced podocyte protection, ultimately delaying the progression of renal injury (Supplementary Appendix Figure 5). This dual inhibition strategy offers new theoretical insights and a promising therapeutic approach for the treatment of lupus nephritis.

## Data Availability

The original contributions presented in the study are included in the article/Supplementary Material, further inquiries can be directed to the corresponding authors.

## Supplementary material

SUPPLEMENTARY FILE S1 Appendices.

SUPPLEMENTARY FILE S2 Uncropped western blots.
