# Supplementary material for: Dual blockade of DPP-4 and CXCL12/CXCR4 axes synergistically protects podocytes in lupus nephritis
Source: Front Pharmacol. 2026 Jan 12;16:1732243. doi: 10.3389/fphar.2025.1732243 (PMC12832377; doi:10.3389/fphar.2025.1732243)

Figure 2B

Uncropped

CXCL12

α-tubulin

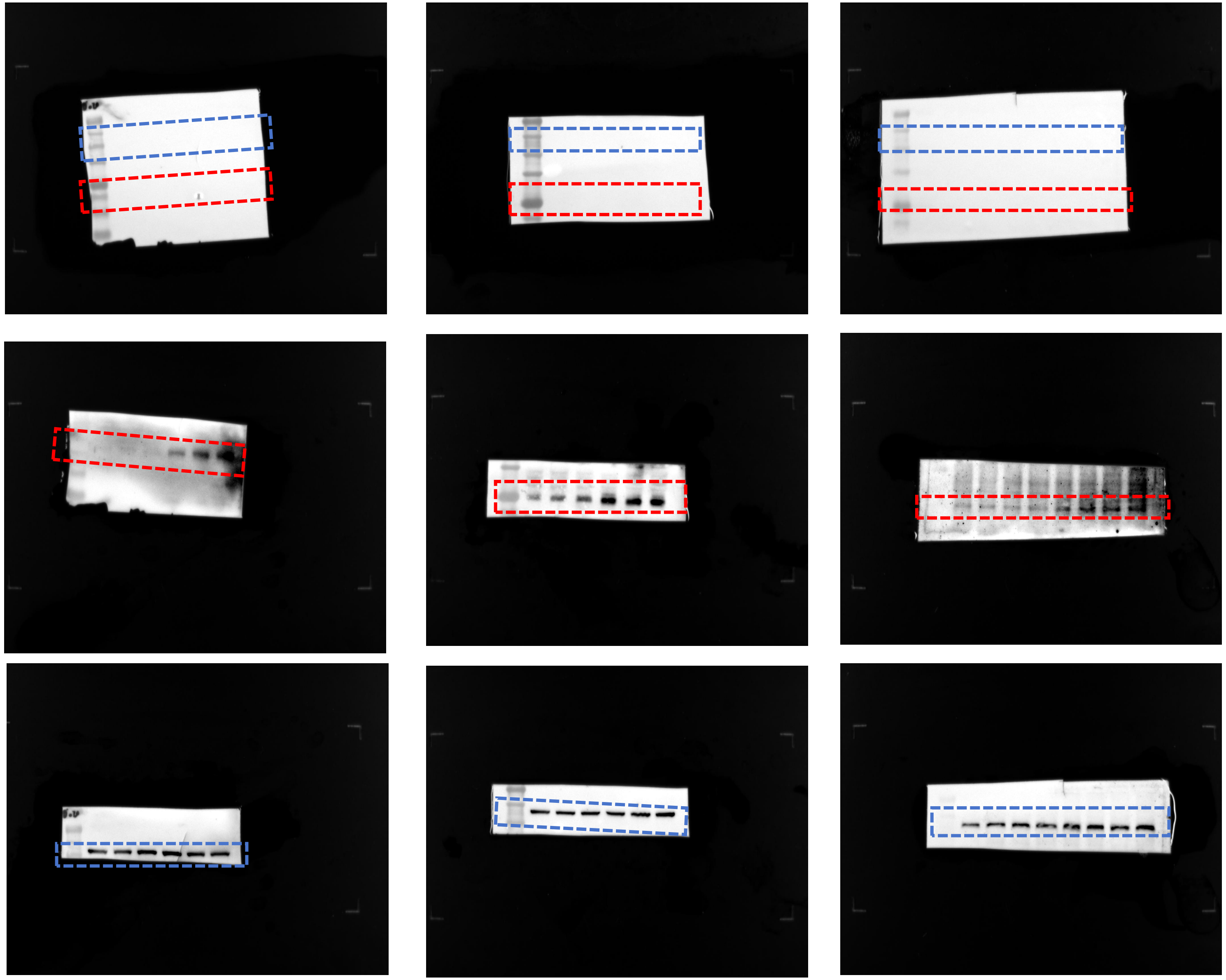

Figure 2C

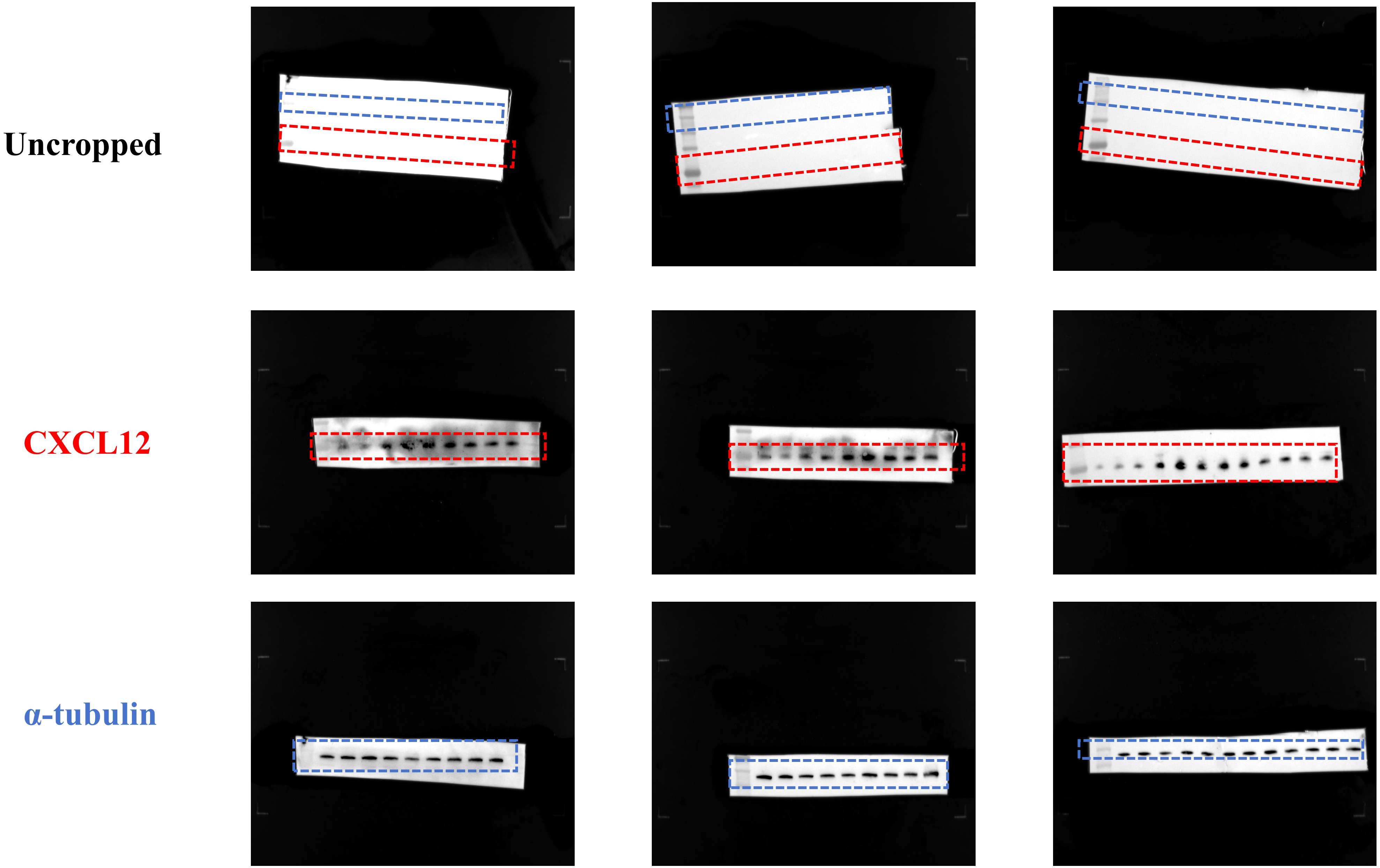

Figure 2C

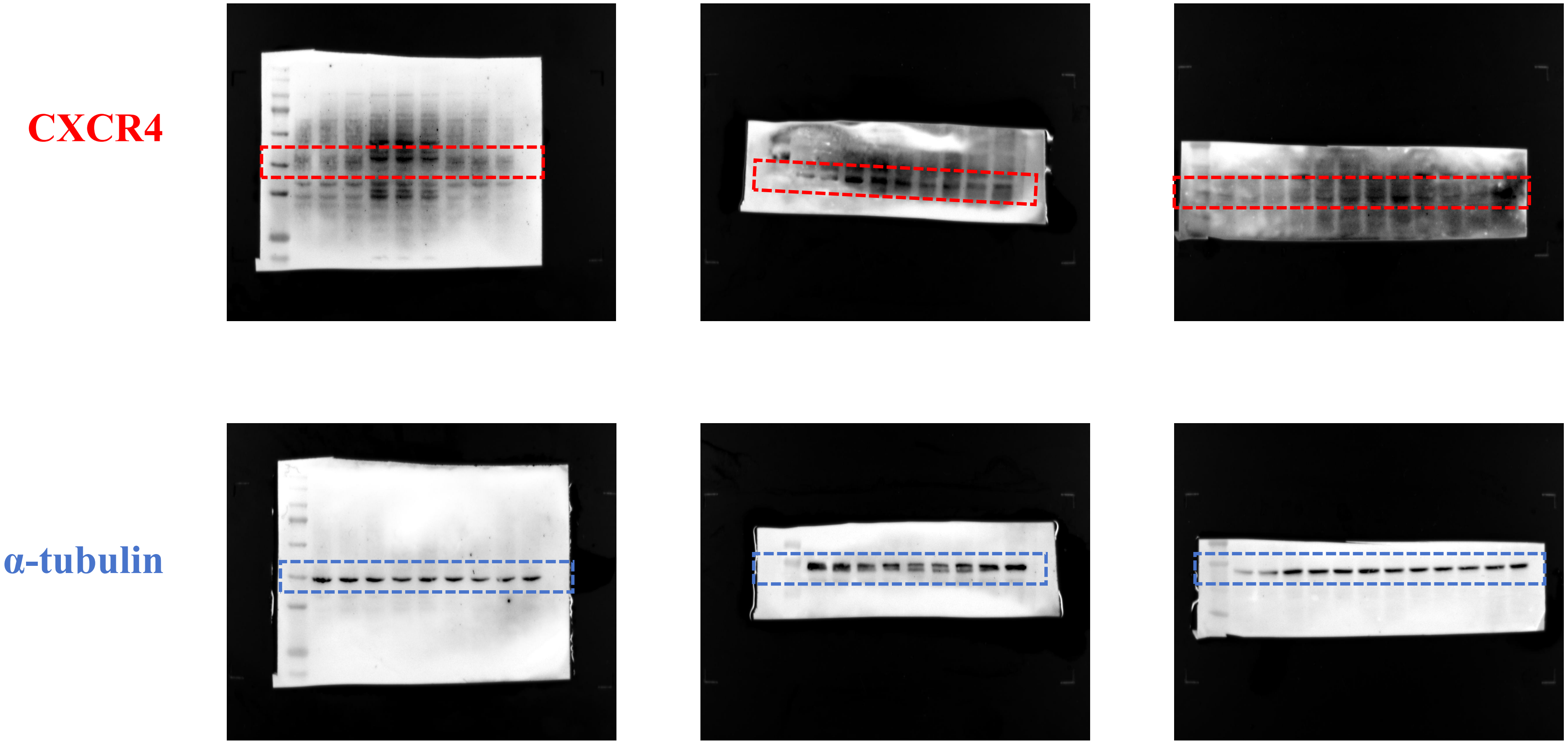

Figure 2C

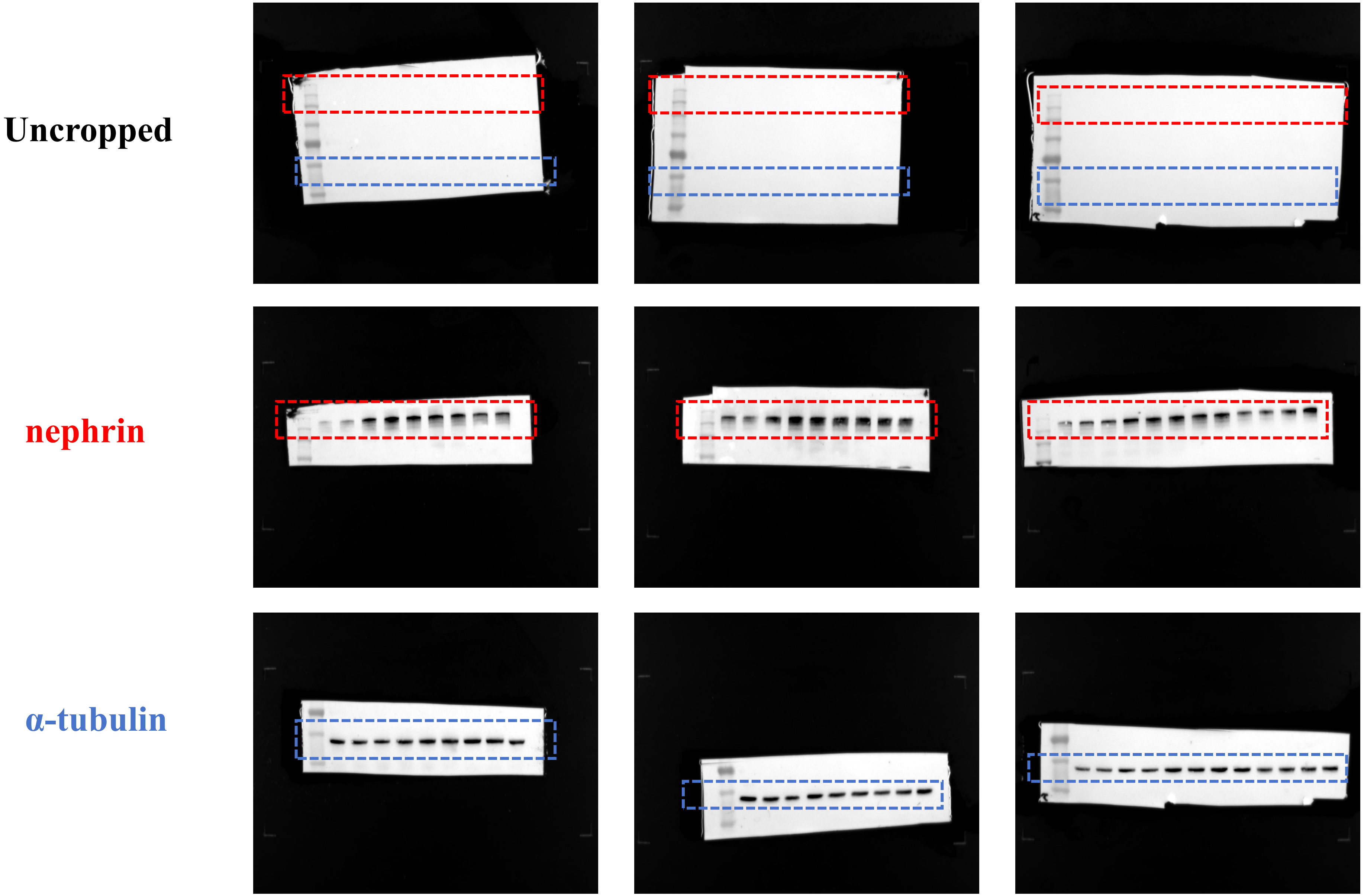

Figure 2D

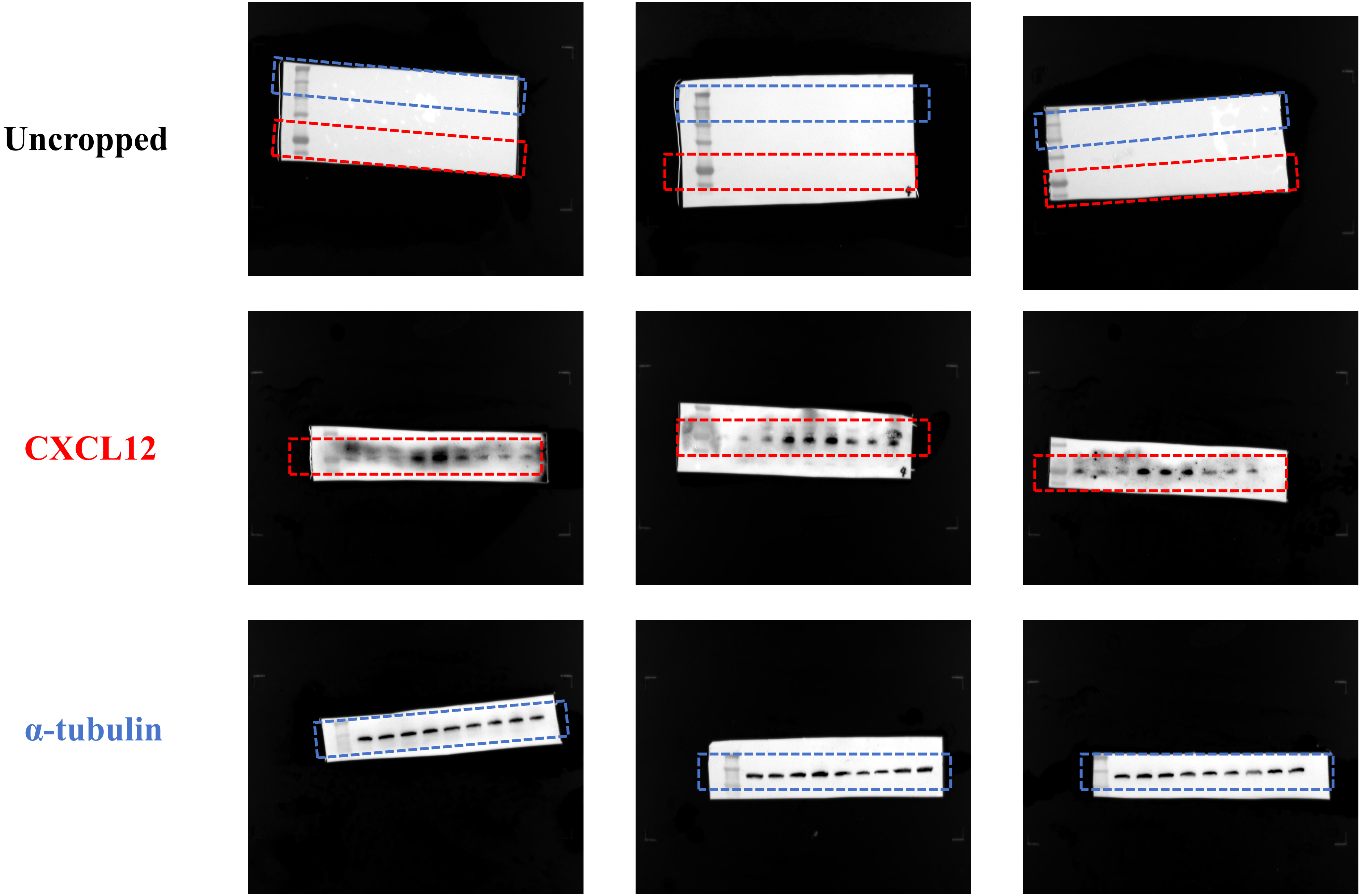

Figure 2D

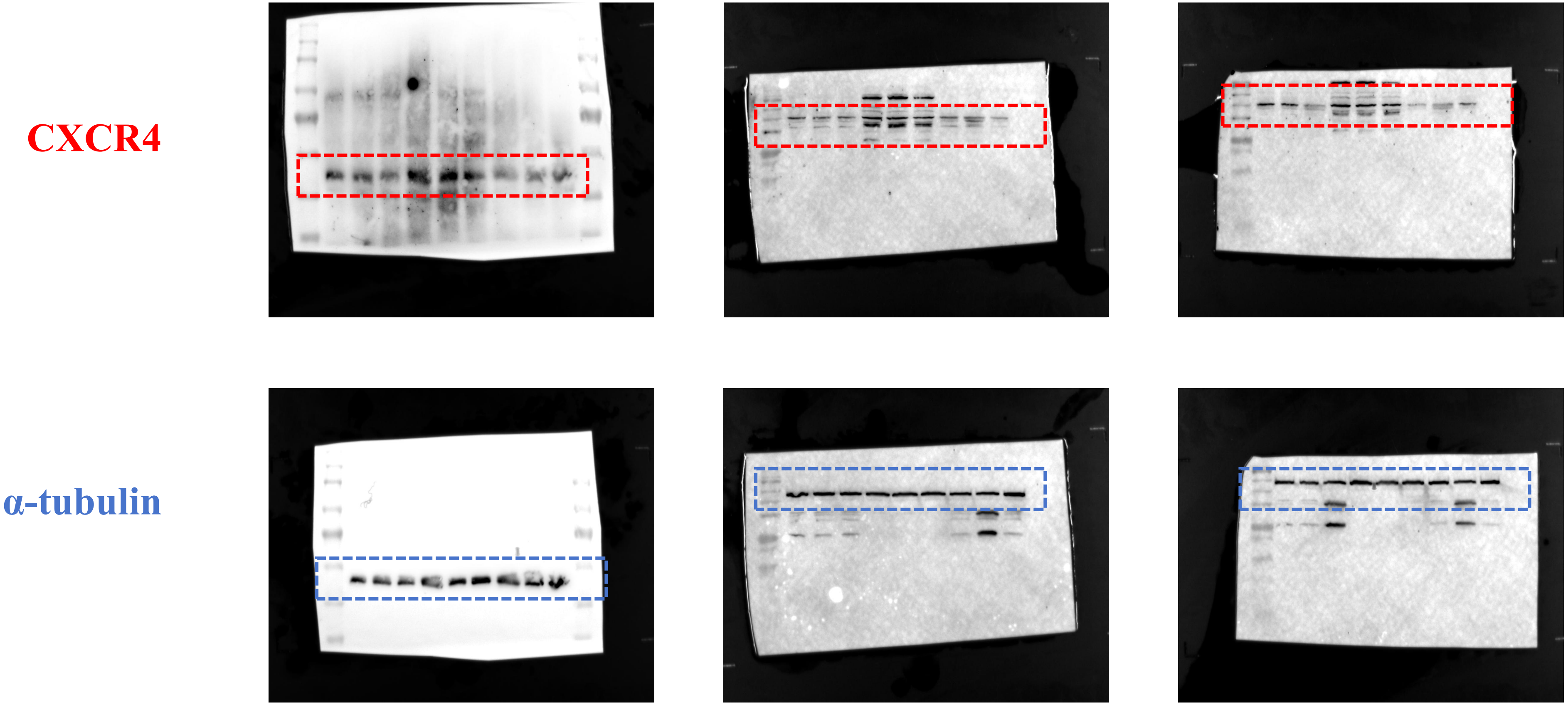

Figure 2D

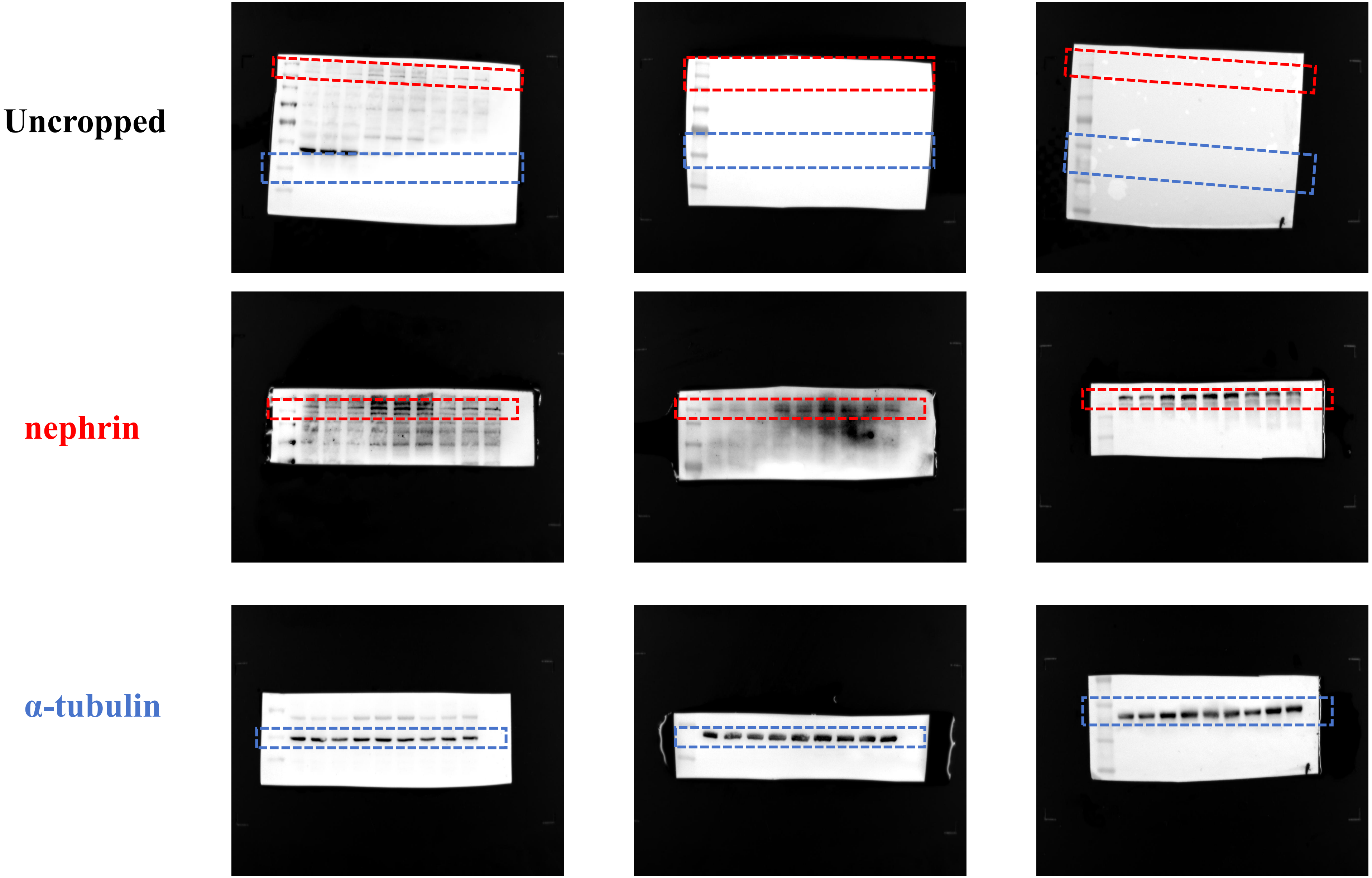

Figure 2E

Uncropped

CXCL12

α-tubulin

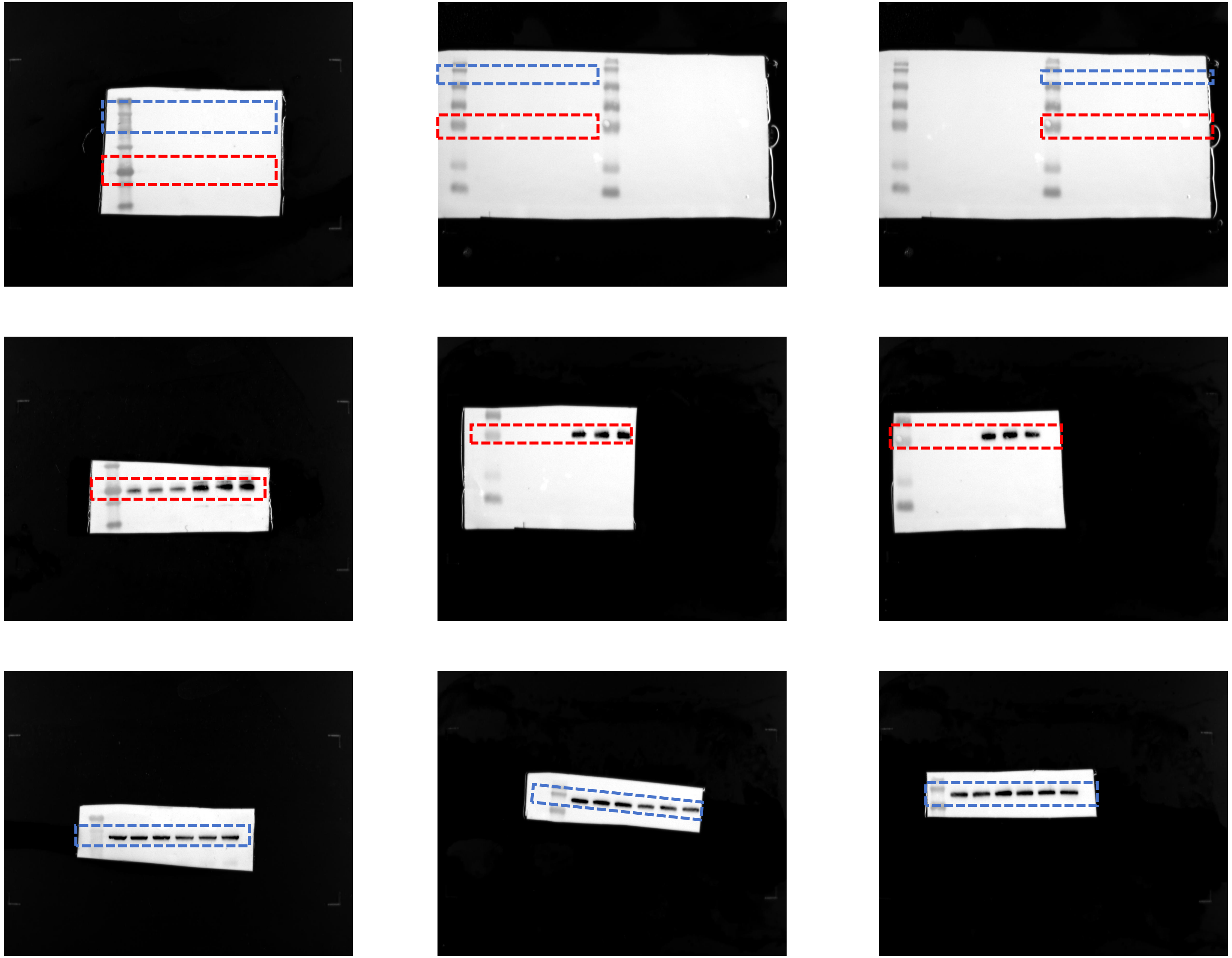

Figure 2E

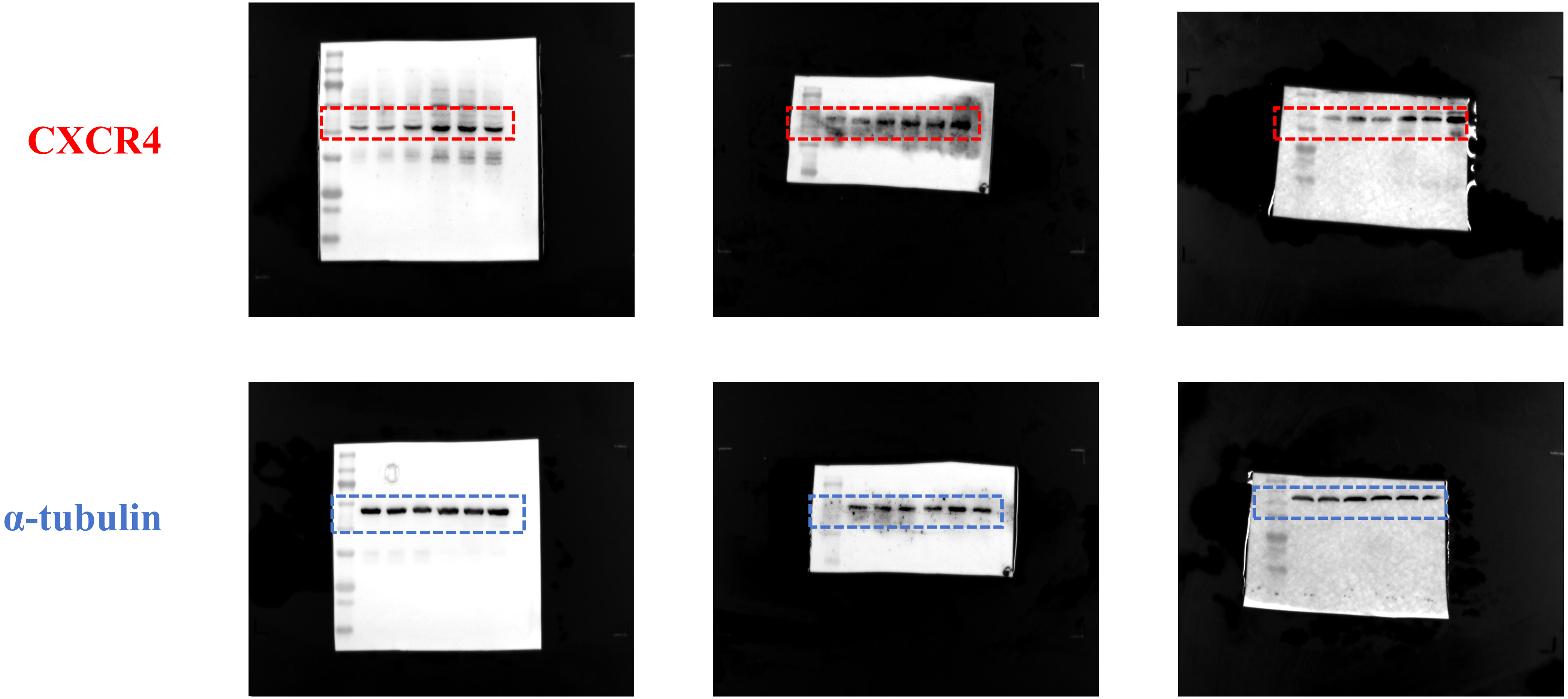

Figure 2E

Uncropped

nephrin

α-tubulin

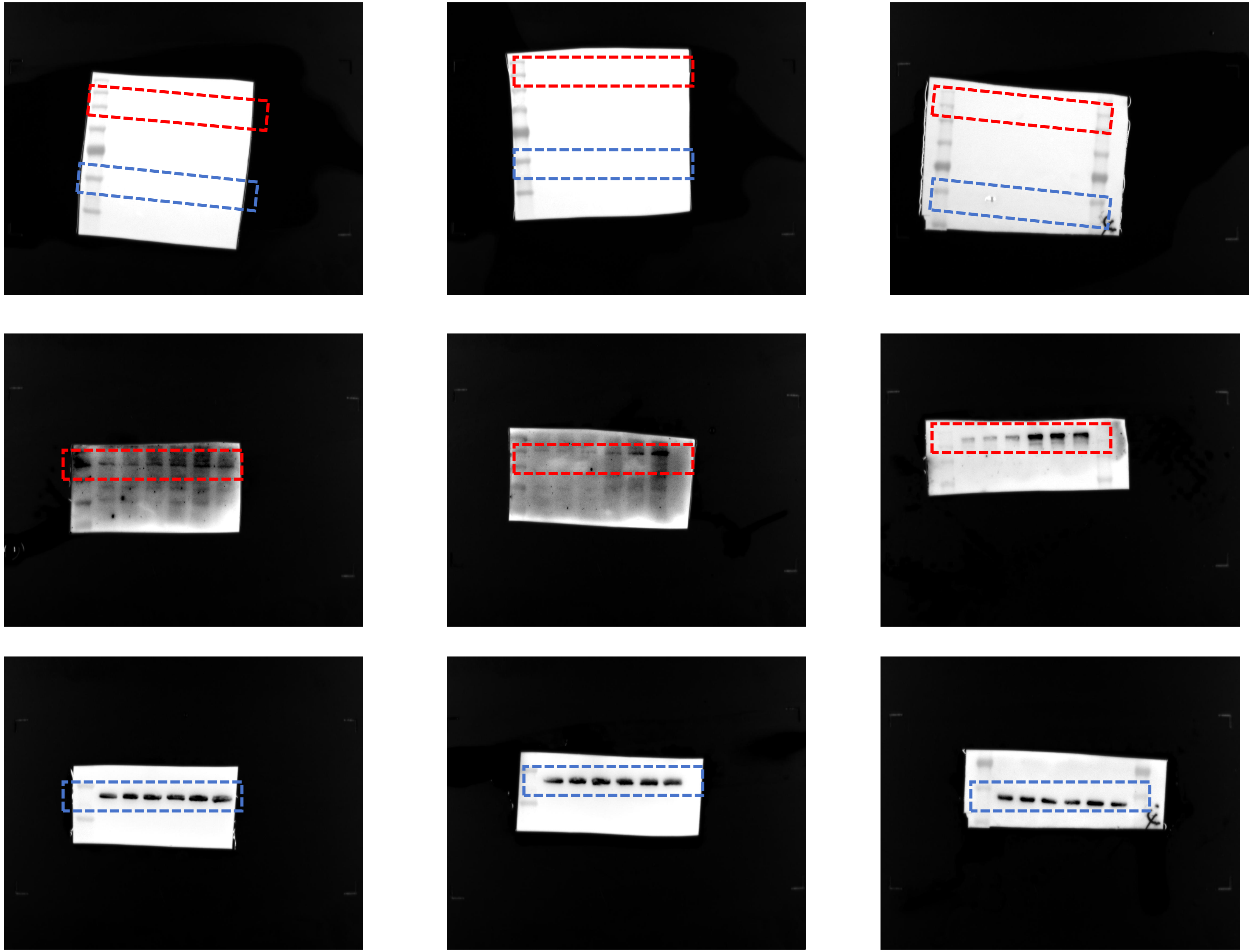

Figure 2F

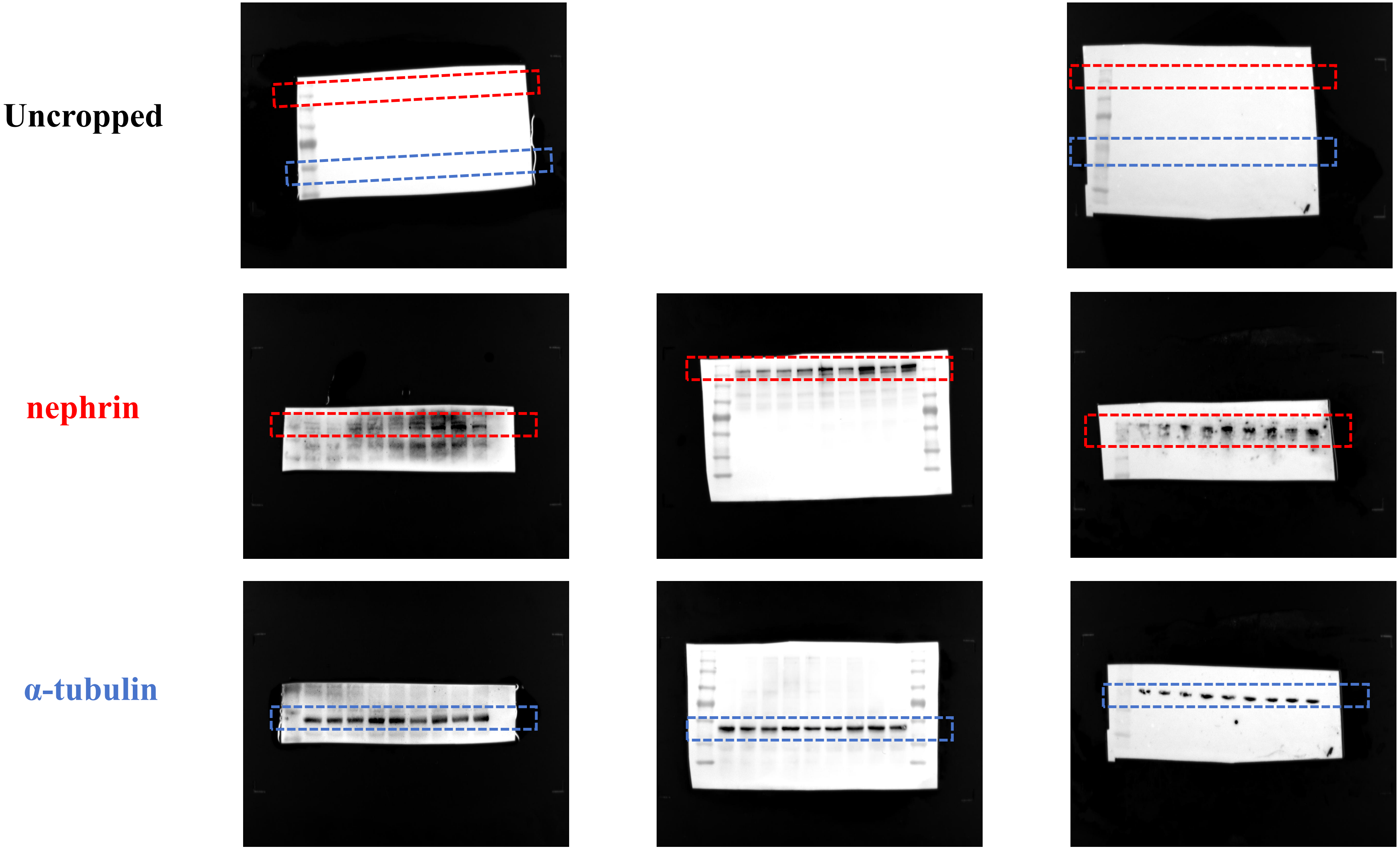

Figure 3F

Uncropped

nephrin

α-tubulin

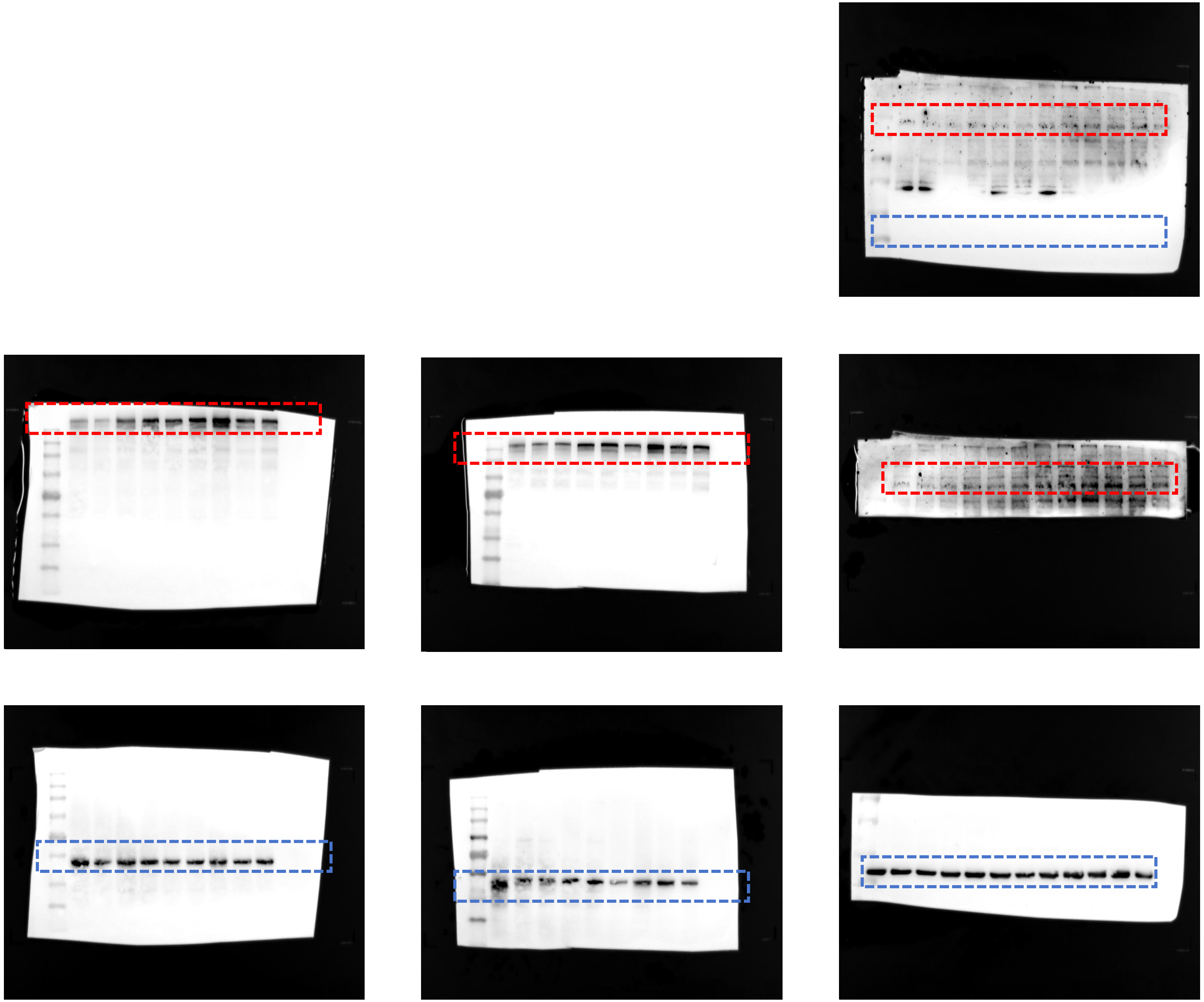

Figure 3F

podocin

α-tubulin

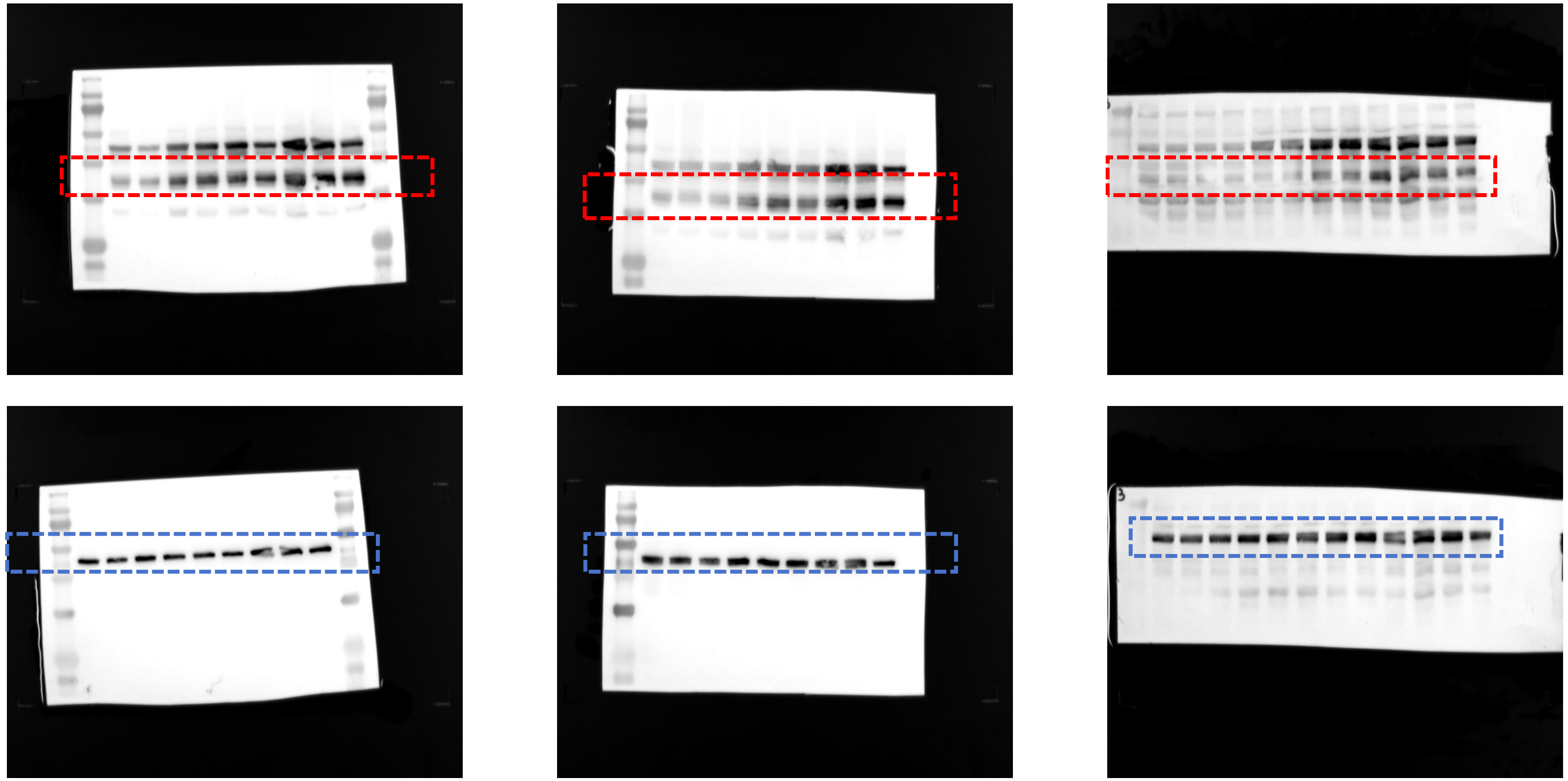

Figure 4B

Uncropped

FN

α-tubulin

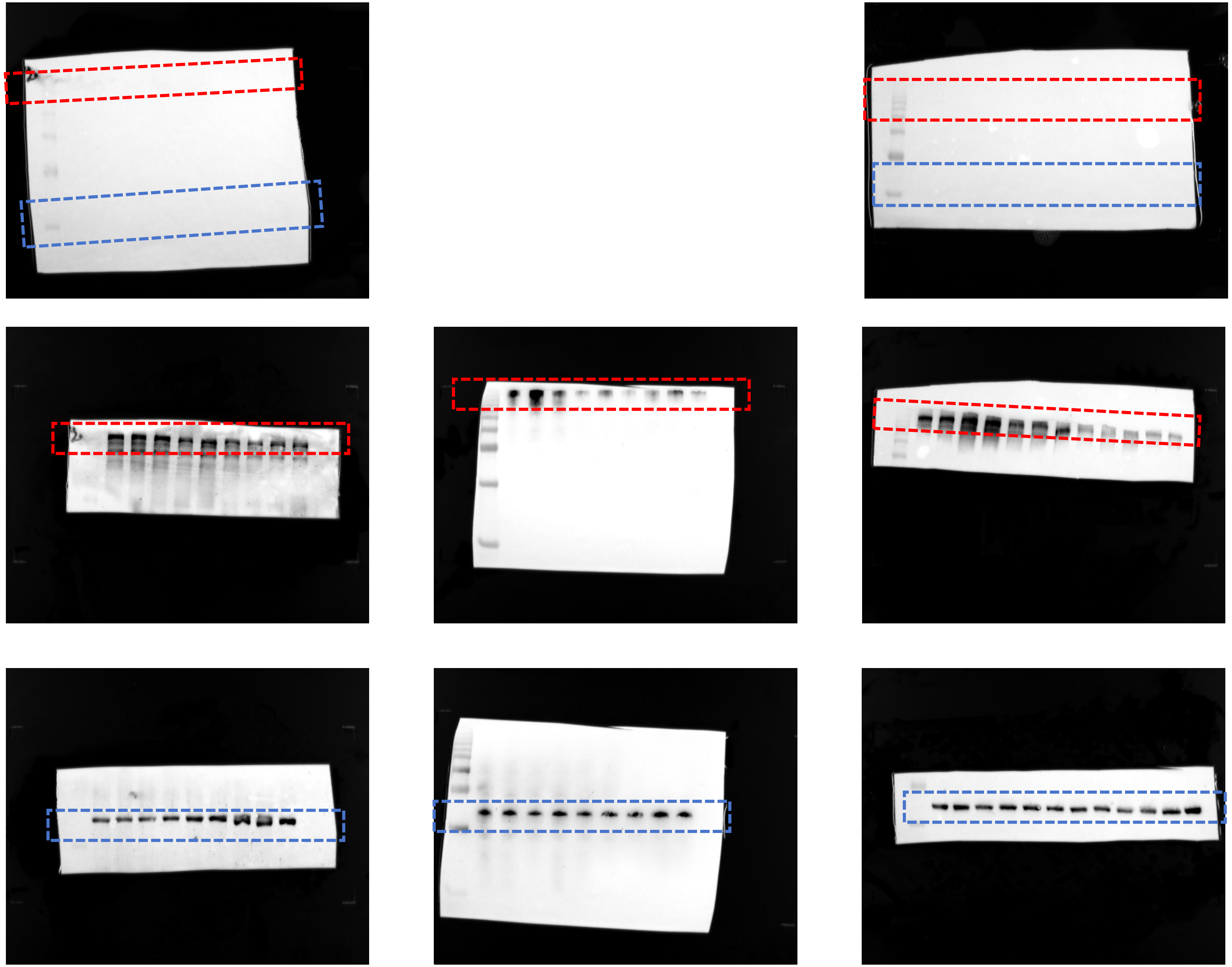

Figure 4B

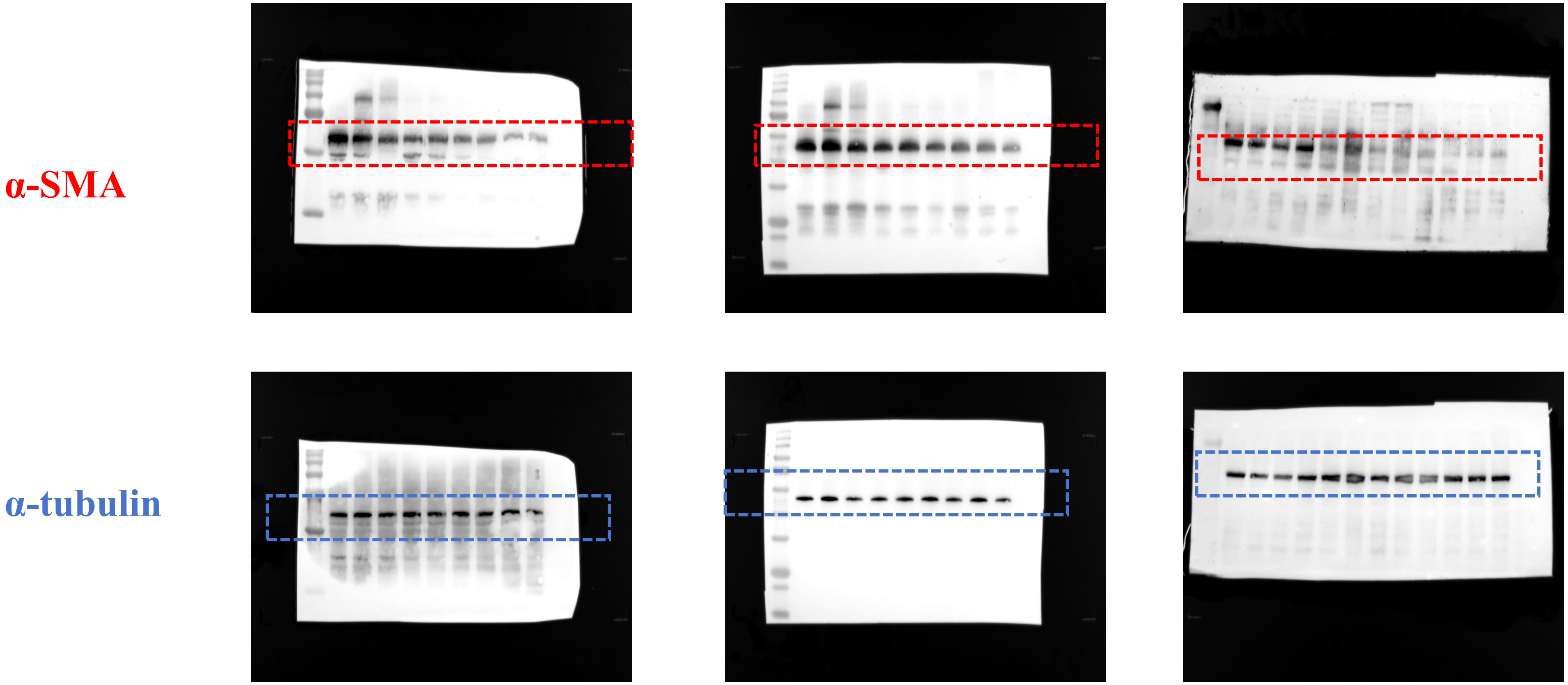

Figure 4C

Uncropped

NLRP3

α-tubulin

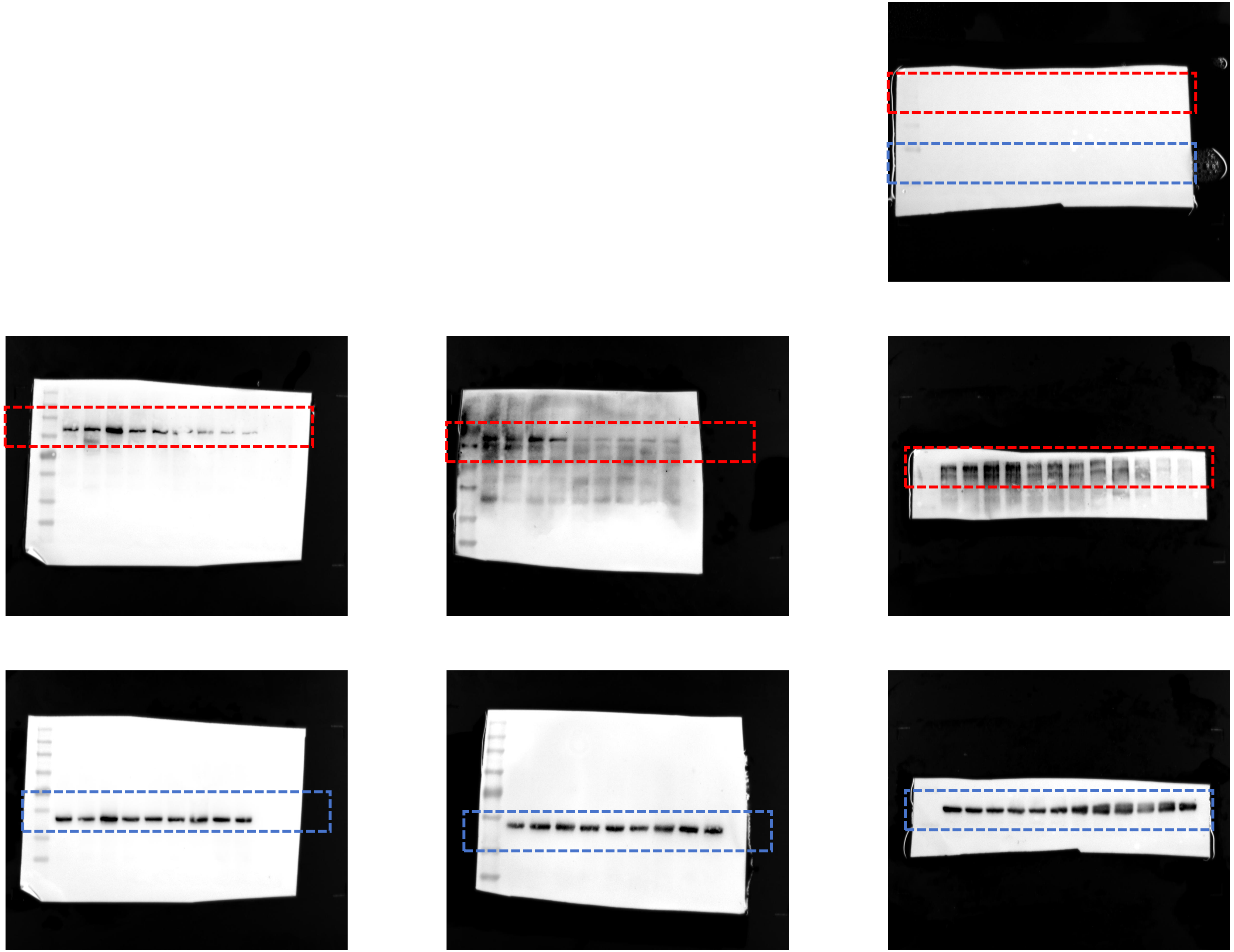

Figure 4C

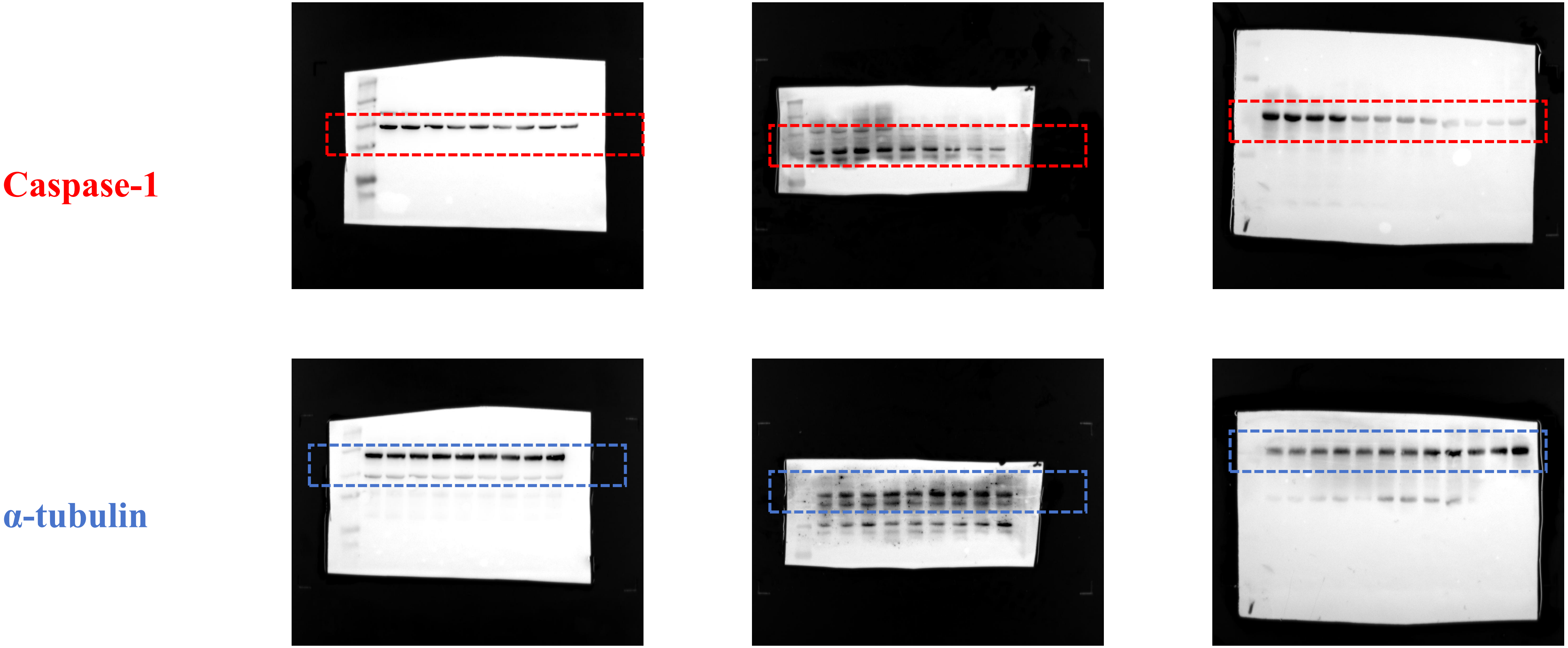

Figure 4C

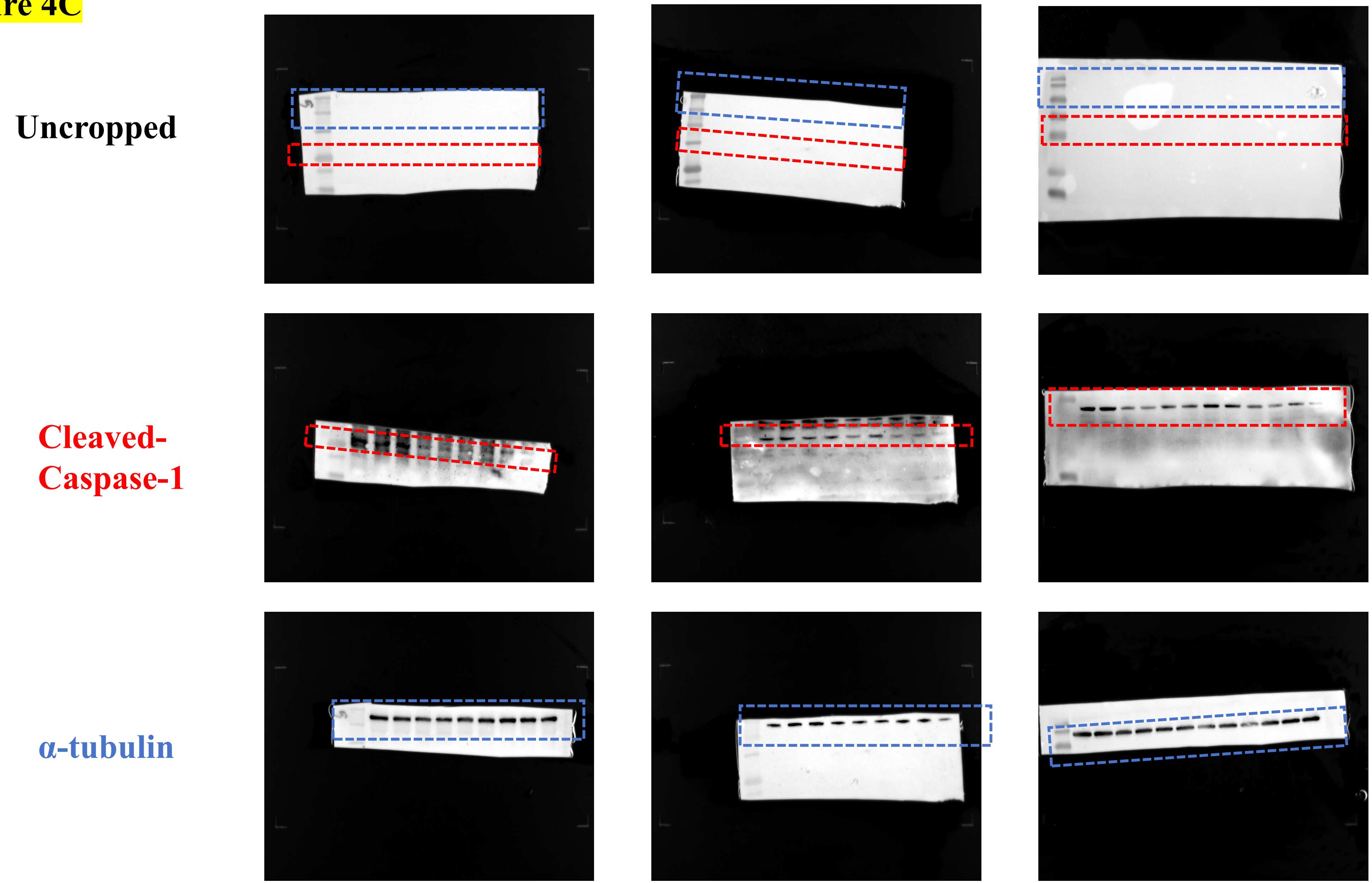

Figure 4C

IL-1β

α-tubulin

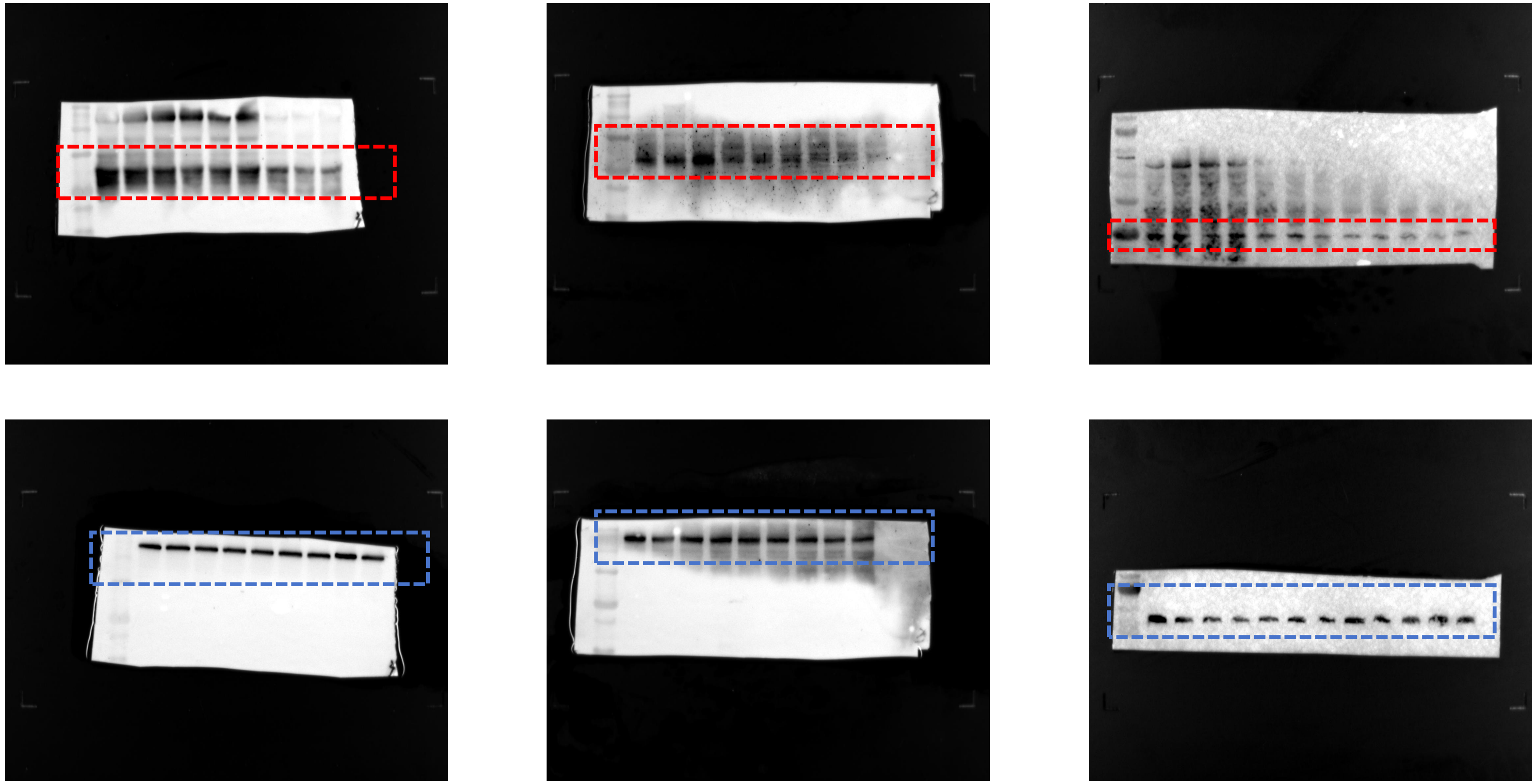

Supplement: Supplementary file 1 [file DataSheet1.pdf]
